# The role of epigenetics in T-cell lymphoma

**DOI:** 10.1007/s12185-022-03470-1

**Published:** 2022-10-14

**Authors:** Makoto Yamagishi

**Affiliations:** Laboratory of Tumor Cell Biology, Department of Computational Biology and Medical Sciences, Graduate School of Frontier Sciences, The University of Tokyo, 4-6-1, Shirokanedai, Minato-ku, Tokyo, 108-8639 Japan

**Keywords:** Epigenome, Histone modification, DNA methylation, EZH2

## Abstract

Malignant lymphomas are a group of diseases with epigenomic abnormalities fundamental to pathogenesis and pathophysiology. They are characterized by a high frequency of abnormalities related to DNA methylation regulators (DNMT3A, TET2, IDH2, etc.) and histone modifiers (EZH2, HDAC, KMT2D/MLL2, CREBBP, EP300, etc.). These epigenomic abnormalities directly amplify malignant clones. They also originate from a hematopoietic stem cell-derived cell lineage triggered by epigenomic changes. These characteristics are linked to their high affinity for epigenomic therapies. Hematology has led disease epigenetics in the areas of basic research, clinical research, and drug discovery. However, epigenomic regulation is generally recognized as a complex system, and gaps exist between basic and clinical research. To provide an overview of the status and importance of epigenomic abnormalities in malignant lymphoma, this review first summarizes the concept and essential importance of the epigenome, then outlines the current status and future outlook of epigenomic abnormalities in malignant lymphomas.

## Introduction

Recent large-scale comprehensive analyses have revealed genetic abnormalities and abnormal gene expression in malignant lymphomas, leading to a better understanding of the characteristics and pathogenic mechanisms underlying each disease, and highlighting further research toward stratification to achieve appropriate treatment and diagnosis. However, at the same time, the importance of the epigenome emerges again both from the genome and transcriptome, involving spatiotemporal gene regulation. In particular, multiple non-Hodgkin’s lymphomas accumulate genetic mutations and copy number aberrations in key signaling pathway components, resulting in altered transcription factor activity unique to each lymphoma. However, the expression levels, gene regions, and timing regulated by these transcription factors are all under the control of the epigenome. Furthermore, somatic mutations accumulate in the genes themselves that encode chromatin regulators. It is obvious that epigenomic abnormalities are deeply involved in tumorigenesis. As determinants of reversible gene regulatory networks, epigenomic studies are the most central topic.

The information gathered in the field of epigenomic abnormalities is vast. Listing individual research results makes reviewing the position and importance of “epigenomic abnormalities in malignant lymphoma” difficult. Currently, new epigenetic drugs are being constantly introduced, we consider rediscussing the concept and significance of epigenetic abnormalities an important topic. Therefore, this review aims to summarize the concept and essential importance of epigenetics, then discuss the current status and future perspectives in the field of epigenomic research in malignant lymphoma.

## Intrinsic importance of epigenomic regulation

Clonal cell growth, the underlying cause of malignancy, is triggered by genomic aberrations that are irreversible changes in the genetic material itself, representing a discontinuous process that alters cellular characteristics. The emerging mutant clones are selected based on natural selection, determining the population composition of the upcoming generation.

However, epigenetics is defined as “stably heritable phenotype resulting from changes in a chromosome without alterations in the DNA sequence” [1]. In a broad sense, epigenetics is interpreted as all heritable changes that do not involve changes in the DNA sequence. Nevertheless, in various situations, epigenetics refers to a series of molecular processes involving the chromatin structure and its regulators at the molecular level that actually drives, maintains, and inherits epigenetic processes. Epigenetic processes in the narrow sense include DNA methylation, chemical histone modifications, as well as chromatin structure, and transcriptional regulation by a group of transcription factors.

**Fig. 1:**
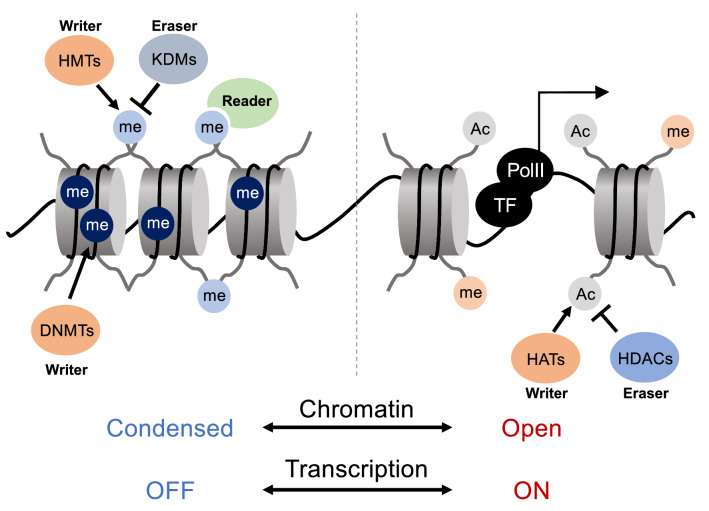
Process of chromatin regulation. Modifying DNA and histones by epigenetic writers, erasers, readers, etc., determines chromatin structure. Relaxation of chromatin structure defines the basal transcription level of the genes

Each of the more than 20,000 genes encoded on DNA is under the control of the chromatin structure. Simply expressed, epigenetic processes determine the “timing” and “amount” of gene expression (Fig. 1). With the progress in molecular biology, individual gene functions became clarified. In parallel, the importance of methylated DNA and histone modifications in gene regulatory processes attracted growing attention, accelerating research in the field of epigenetics. Subsequent advances in analytical techniques have established research methods for genome-wide (epigenome) analysis of methylated DNA, histone modifications, open chromatin structures, etc., establishing that the epigenome is deeply involved in all biological events, such as cell development, differentiation, proliferation, and functional expression.

In addition, mutations, structural alterations, copy number abnormalities, and epigenome regulation-related gene expression level changes were successively identified in various neoplastic, genetic, and inflammatory diseases. Comparative epigenome studies of normal and diseased individuals have also demonstrated large-scale changes in the chromatin structure in the case of disease. Therefore, the importance of the epigenome in influencing a wide range of genes is now widely recognized.

## Epigenomic regulation as a network

There are three main reasons behind the complexity of epigenomic regulation.

**Fig. 2:**
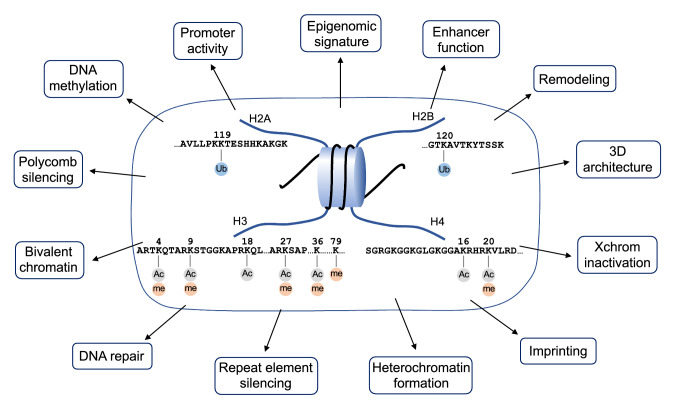
Post-translational modification of histone molecules. Various chemical modifications of the histone N-terminal amino acid residues define the local chromatin structure. Gene expression patterns due to chromatin changes are involved in various cellular processes. This figure includes only those modifications that have demonstrated to be significantly important to the functions

First, the diversity of chemical modifications and regulators: each of the four histone molecules displays various amino acid residues such as lysine, serine, and arginine at its N-terminus. Each of them undergoes various chemical modifications (e.g., methylation, acetylation, phosphorylation, ubiquitination), conferring diverse biological significances (Fig. 2) [2]. For example, histone H3 lysine residue trimethylation (me3) at positions 4 (H3K4), 9 (H3K9), and 27 (H3K27) from the N-terminus exhibit different biological significances, roughly classified as H3K4me3 that positively regulates transcription, as well as H3K9me3 and H3K27me3 that negatively regulate transcription. H3K27me3 functions mainly as a repressive marker in euchromatin regions with high gene activity levels.

Independent factors are also involved in these modifications. Factors involved in chromatin regulation are classified according to their functions (e.g., Writer, Eraser, Reader, Remodeler, Recruiter). The organization of post-translational histone modifications is complex, involving multiple factors. For example, both the H3K4 (Trithorax) and the H3K27me3 (Polycomb) methyltransferase complexes function genome-wide to form complex modification patterns, and factors recognizing them determine the chromatin structure of the corresponding regions (Fig. 1) [3]. Furthermore, the activity levels of the multiple transcription factors that are accessed allow precise and high-resolution gene regulation.

Second, it is necessary to have a spatiotemporal perspective: chemical DNA and histone modifications are not quantitatively equal to gene expression. The location of the modification site and the genetic region are important parameters. For example, repression marks (*e.g.*, methylated DNA or H3K27me3) located far from the transcription start site (TSS) affect gene expression differently than those located near the TSS. Therefore, the relationship between the activity of a particular epigenetic regulatory system and a group of target genes is not uniformly defined [4].

In higher organisms (*e.g.*, mammals), no information is encoded in the DNA sequence (at least not deciphered to date) as to which regions undergo epigenetic modification, but rather Recruiters recognize the chromatin structure and other modification patterns and recruit the modifying enzymes to a specific genomic region. This is the driving mechanism that generates diverse expression patterns from the same DNA sequence, allowing for complex development, differentiation, and growth regulation.

For example, DNA methylation patterns reportedly correlate with the H3K36me2/3 mark [5]. It is also known to correlate with the H3K27me3 pattern, but it seems to vary context-dependently [6, 7]. Epigenomic modifications are difficult to predict. Instead, techniques that allow the genome-wide analysis of epigenetic modifications, by such methods as ChIP-sequencing, have been established and generalized.

Third, gene regulation forms a complex network. Mutations and expression changes in epigenetic process-related factors serve a genome-wide function and affect hundreds to thousands of target genes. The regulated genes also include multiple transcription and signaling pathway factors, forming an even more complex secondary gene regulatory network. The gene expression changes resulting from aberrations in the epigenomic factors are extremely vast.

Epigenome-related factors act upstream in the gene network hierarchy, which is the essence of epigenomic regulation in cell fate determination [8]. When understanding epigenomic abnormalities, it is critical to consider their effects on individual genes and, at the same time, to view them as a network from a macro perspective. Qualitative and quantitative changes in epigenome-related factors have genome-wide effects and alter the network itself. Furthermore, the upcoming generation of cells inherits these changes through complex steps, which is highly relevant to malignant tumor characteristics.

## Epigenomic abnormalities as therapeutic targets

Epigenetic changes would converge on the chromatin structure of individual gene loci. Since chromatin structure defines the basic amount of gene expression, the general direction in which cells are orientated is known to correlate well with chromatin structure patterns. Compared to gene expression patterns, which are often noisy, open chromatin structure data obtained by DNase-seq, ATAC-seq, and other methods provide a good indication of the cell differentiation stages [9]. In contrast, genome-wide changes in the chromatin structure indicate a significant variance from normal.

A particularly notable epigenomic regulation property from a therapeutic perspective is the continuity and reversibility of the epigenetic responses. This characteristic is often used in contrast to genomic abnormalities. Methylated DNA and various histone modifications, representing the molecular level of the epigenome, are determined by continuous equilibrium reactions controlled by modifying enzymes and cofactors. Therefore, it is theoretically possible to shift the equilibrium to normal by externally adjusting enzymatic activity with small-molecule compounds.

Cell proliferation, inflammation, immortalization, and escape from tumor immunity are also driven by genetic abnormalities that accumulate in tumor cells. The expression levels, target regions, and timing of the gene clusters that shape their molecular pathogenesis are all under epigenomic control. Epigenome regulation might also lead to driver gene function control. Therefore, reconsidering its influence, epigenomic regulation is extremely promising as a therapeutic target and is indeed effective, at least in the case of hematological malignancies.

## The vulnerability of epigenomic regulation based on abnormal patterns

It is useful to organize the abnormal epigenomic factor accumulation patterns to examine the vulnerability of the epigenomic regulatory systems and therapeutic target molecules. In hematologic tumors, the overwhelming majority of the genes with mutations, structural, and expression abnormalities are detected are epigenetic writers such as *DNMT3A, EZH2, KMT2D/MLL2, CREBBP, EP300*, and epigenetic erasers such as *TET2* and *KDM6A/UTX*. Several experiments have shown that these abnormalities lead to genome-wide DNA and histone modification pattern alterations, modify the chromatin structure, and indeed have the potential to cause disease. These facts convincingly indicate that changes in the modifying enzymes themselves have a significant impact on the epigenome, and the development of molecularly targeted therapies based on this mechanism is actively promoted.

However, solid tumors display multiple genetic abnormalities of chromatin remodeling factors, such as those of *ARID1A, ARID1B, SMARCA4,* and *SMARCB1*, which are thought to drive clonal proliferation in different ways. Overexpressed EZH2 and H3K27me3 abnormalities have been reported in various cancer types, although they appeared to be less dependent than in hematological tumors. Tumor-specific aberrant super-enhancer formation in conjunction with signaling pathway and transcription factor activation, such as that of MYC, along with multiple causative mechanisms have been reported [10, 11]. Multifaceted approaches have been proposed for these epigenomic abnormalities in solid tumors.

## Relevance of epigenomic abnormalities in tumorigenesis

**Fig. 3:**
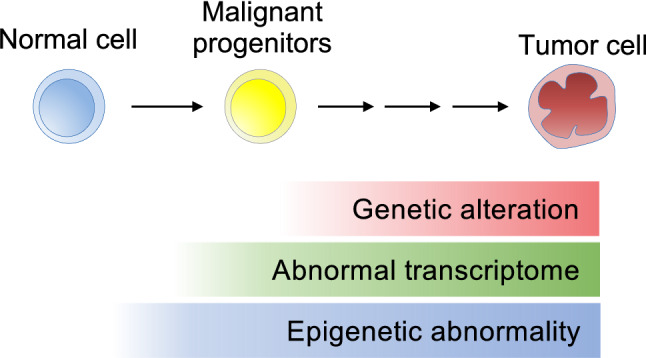
Epigenomic abnormalities are critical in the early stages of the tumorigenic process. Targeting epigenomic abnormalities common to heterogeneous tumor cell populations is expected to achieve a durable therapeutic effect and early therapeutic intervention

Another important feature of epigenomic aberrations is their early involvement in tumorigenesis. As several studies on cancer development showed [12], epigenomic aberrations could be detected in cells in a precancerous state and might function as fundamental molecular abnormalities from which malignant clones evolve (Fig. 3).

It is supported by various hematological observations. In clonal hematopoiesis of indeterminate potential (CHIP), epigenome-related gene abnormalities are central and correlate with the risk of developing MDS and AML [13]. Interestingly, in cases that progress to secondary AML, mutations in signaling pathway activator *FLT3, WT1, NMP1*, and *NRAS* are found in clones with mutations in *TET2, DNMT3A*, etc. The accumulation of driver mutations that induce malignant transformation in clones with epigenomic abnormalities is considered to be a risk factor for disease development.

Loss-of-function (LOF) mutations in the *TET2* gene, which are frequently detected in peripheral T-cell lymphoma (PTCL), have a relatively high variant allele frequency compared to other mutations in the same patients and are considered to be an early event in tumorigenesis [14]. *EZH2* mutations in follicular lymphoma (FL) and diffuse large B-cell lymphoma (DLBCL) (see below) are reportedly also early abnormalities [15].

Early epigenomic abnormalities in adult T-cell leukemia-lymphoma (ATL) have also been identified: single-cell analysis of tumor clones in several cases that progressed from HTLV-1-infected carriers to acute ATL via smoldering ATL revealed that in a multistep tumorigenesis process H3K27me3 accumulated genome-wide from an early stage [16, 17]. Some of these epigenomic aberrations can also be detected in polyclonal pre-onset carrier populations and before progression to acute ATL [18, 19]. Targeting common epigenomic abnormalities in heterogeneous tumor cell populations due to diverse genomic architecture could lead to sustained therapeutic efficacy and early therapeutic intervention.

## Hematologic tumors and epigenomic abnormalities

Myeloid and lymphoid tumors are more likely to respond to epigenomic therapy than solid tumors. Therefore, several therapeutic agents have been approved after a number of clinical trials. How could this clinically experienced phenomenon be explained?

**Fig. 4:**
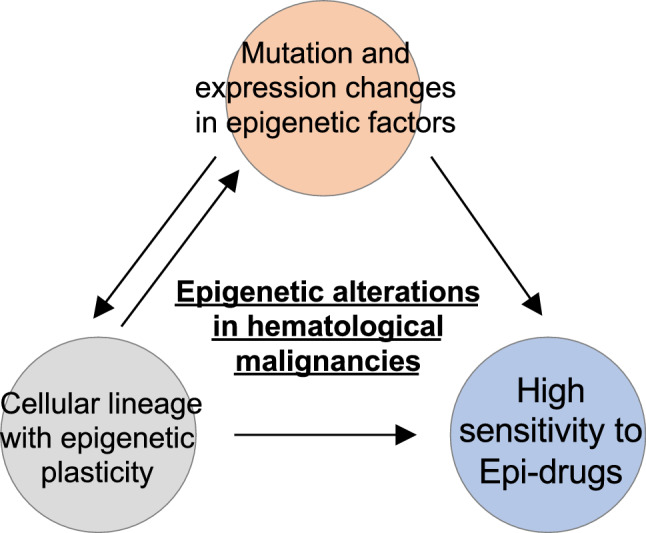
Characteristics of hematologic tumors correlate with the efficacy of epigenomic therapies

Hematological malignancies are characterized by (1) a high frequency of DNA methylation regulator aberrations (e.g., those of *DNMT3A, TET2, IDH1/2*) and histone modifiers (e.g., those of *EZH2, KMT2D/MLL2,* and *CREBBP*), and are directly driven by epigenomic abnormalities, and (2) the epigenomic alteration-related hematopoietic stem cell-derived lineage is the cell of origin and highly permissive to epigenomic alterations. These characteristics are consistent with their high affinity for epigenetic drugs (epi-drugs) (Fig. 4).

## Epigenomic abnormalities in T-cell lymphoma

PTCL is a highly malignant and heterogeneous group of diseases with aggressive disease course and poor clinical outcomes. Epigenomic abnormalities are deeply involved in disease pathogenesis and mechanisms (Table 1) [20].

**Table 1:** Genetic abnormalities of epigenetic regulators in T-cell lymphoma

Mutation gene	Subtype	Frequency	Frequent mutation in tumor type	
TET2	AITL	42–89%	MPN	~ 13%
	PTCL-NOS	28–48%	CMML	~ 50%
			MDS	25%
			AML	23%
DNMT3A	AITL	25–33%	AML	20–30%
	PTCL-NOS	27%	MDS	10–15%
IDH2	AITL	20–45%	AML	8–19%
	PTCL-NOS	8%	MDS	~ 5%
KMT2D	AITL	25%	DLBCL	35–85%
	PTCL-NOS	36%	FL	89%
SETD2	EATL	32%	Renal cell carcinoma	13–30%
	MEITL	91%		

The frequencies of mutations were referred to in Reference [20]

Mutations in *TET2* and *IDH2* are the most frequent, and abnormalities in methylated DNA are closely associated with the pathogenesis. *TET2* is a tumor suppressor gene, catalyzing DNA demethylation, LOF mutations are particularly common in follicular helper T-cell (Tfh) lymphomas, and *TET2* deficiency might contribute to the formation of AITL and Tfh-like lymphomas [21]. In addition, *IDH2* R172 gain-of-function mutations, frequently found in AITL, cause DNA methylation induction and a increase in histone H3K27me3 levels by inhibiting the TET2 enzyme catalytic activity [22].

These abnormalities might affect the efficacy of DNMT inhibitors such as 5-azacytidine and decitabine. Several clinical studies suggested that *TET2* mutation cases show a higher response rate compared to wild-type cases [23–25]. However, due to the limited number of cases and genotypes, it has not been established as an accurate biomarker. Effector mechanism clarification and large-scale clinical trials are required to determine whether *TET2* or *IDH2* genetic abnormalities could provide a rationale for using DNMT inhibitors.

PTCL and cutaneous T-cell lymphoma (CTCL) are also disease groups with effective HDAC inhibitors, empirically interpreted as HDAC activation. Although HDACs form a large subfamily, various HDAC molecules are overexpressed in PTCL. HDAC inhibitors reportedly induce cell cycle arrest, apoptosis, and DNA damage, to regulate multiple intracellular pathways [26], transform chromatin structure, and regulate transcription factor activity [27], but the mechanisms of antitumor effects and drug resistance remain unclear. Further studies would be required to determine the effector mechanisms and biomarkers underlying HDAC inhibitor efficacy in treating these diseases.

## Emerging therapies targeting H3K27me3 abnormalities

H3K27me3 is a key histone modification responsible for the repression of highly active euchromatin regions and is dynamically altered during the normal-to-tumor transition, making it particularly significant in the malignant lymphoma pathogenesis and epigenomic therapy. EZH2, a central writer molecule involved in tumorigenesis and maintenance, was observed with genetic abnormalities and aberrant expression in several malignant lymphomas and solid tumors [3, 28].

EZH2 is involved in the cell cycle checkpoint and inhibition of B-cell differentiation induction, and is essential for embryonic center B cells [29, 30]. EZH2 mutations detected in DLBCL and FL, which are heterozygous for the wild-type, cause abnormal H3K27me3 accumulation [31] leading to reduced response to T-cell-inducing signals and differentiation arrest [29]. It reportedly also acts in immune escape via MHC class II gene suppression [32]. Although tumor suppressor gene promoter suppression is aberrantly expanded, the overall picture of EZH2-mediated chromatin dysregulation and its disease-related importance remains unclear.

Epigenomic therapies targeting methylated histones have been intensively investigated and developed for long. Recently, a highly selective oral EZH2 inhibitor (tazemetostat) was shown to be highly effective (overall response rate of 69%) [33] in a single agent phase II study in relapsed and refractory FL with EZH2 mutations. The same results were also obtained in Japan [34], and the drug was approved for use in EZH2 mutant FL in Japan in June 2021. This might indicate the importance of *EZH2* abnormality in FL. On the other hand, overseas studies showed 35% efficacy in *EZH2* wild-type cases. Moreover, although *EZH2* mutations account for only 25% of all the FL cases, EZH2 function is important for GC formation even in wild-type cases [29], and genetic abnormalities, such as *KMT2D, CREBBP*, and *BCL6*, are also accumulated in FL. Taken together, H3K27me3 seems to be a particularly important epigenomic regulation.

EZH1, an EZH2 paralog, also exists as an H3K27me3 enzyme. EZH2 and EZH1 are dominantly expressed in undifferentiated cells and differentiated lymphocytes, respectively, and complement each other in maintaining the homeostasis of methyltransferase complex (PRC2) function. The presence of these two enzymes provides redundancy in H3K27 methylation. Indeed, when EZH2 is inhibited alone, EZH1 compensates for the function of EZH2 [35, 36]. EZH1 and other PRC2 factors are less frequently aberrant in gene and expression compared to EZH2. This could be attributed to the higher activity and plasticity of EZH2, although the details remain unknown. A univariable analysis has reported that high EZH2 and H3K27me3 expression are associated with poor overall survival (OS) and progression-free survival (PFS) in patients with T-cell lymphomas [37].

**Fig. 5:**
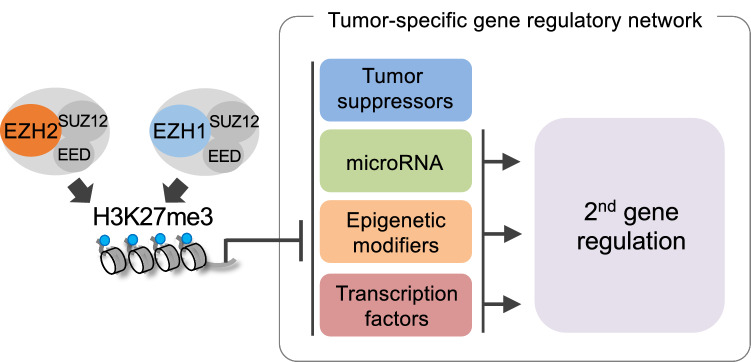
Accumulation of H3K27me3 by EZH1 and EZH2 suppresses the expression of various target genes. It also causes secondary gene expression changes, forming a tumor-specific gene regulatory network

ATL, a T-cell lymphoma with extremely poor prognostic, has an excessive H3K27me3 accumulation [18, 38]. In ATL cells, *EZH2* gene abnormality could not be observed, but activation of NF-κB causes overexpression of EZH2, which maintains abnormal coexistence of the EZH1 or EZH2 methylation complexes and cooperatively causes H3K27me3 accumulation [19] (Fig. 5). Since there EZH1 functions dominantly in the case of multiple target genes, targeting not only EZH2 but also EZH1 would be necessary to regulate genome-wide H3K27me3 accumulation. The fact that H3K27me3 accumulates in correlation with EZH1/2 binding and affects overall gene expression also indicates the importance of their catalytic activities and function as a PRC2 complex.

A new dual inhibitor against EZH1 and EZH2 (valemetostat) is effective against several malignant lymphomas, including ATL [19]. Valemetostat eliminates H3K27me3 more effectively than EZH2-selective inhibitors and dramatically restores target gene expression. The EZH1/2 dual inhibitor also exhibited a pronounced effect on cell proliferation; it was effective at concentrations less than one-hundredth of those of EZH2 selective inhibitors. Interestingly, both EZH1 and EZH2 are functional not only in multiple lymphomas with wild-type EZH2 but also in certain B-cell lymphomas with heterozygous mutant EZH2, and the dual inhibitor is more effective. A Japanese phase II clinical trial (NCT04102150) showed a 48% response rate (CR 20%) as a single agent in 25 patients with relapsed and refractory ATL, confirming the promising efficacy and an acceptable safety profile for patients with relapsed/refractory ATL [39]. Valemetostat has also been evaluated in patients with AITL and PTCL not otherwise specified (PTCL-NOS) in a phase I clinical trial (NCT02732275), suggesting potential clinical benefits.

Therefore, targeting both EZH1 and EZH2 could reverse H3K27me3 abnormalities more effectively. In addition, genetic mutations in epigenetic factors such as the SWI/SNF complex, frequently observed in various cancer types, cause H3K27me3 accumulation by EZH1 and EZH2, and dual inhibitors might be effective in these cases as well [19, 40]. In addition, preclinical studies have suggested that EZH1/EZH2 inhibition can selectively eliminate malignant progenitor cells, such as HTLV-1-infected cells [19, 41]. In aiming for early therapeutic intervention for diseases with poor prognosis, new therapeutic strategies targeting epigenomic abnormalities at the basis of the tumor are expected to be expanded.

## Future perspectives

Biomarkers and drug resistance are major challenges in achieving epigenome-targeting precision medicine. In addition, as exemplified by EZH2, the functions and roles of epigenomic regulatory systems significantly differ depending on the differentiation stage and cell lineage. A basic understanding of epigenomic regulation would be essential to improve therapeutic efficacy, persistence, and safety. Epigenomic heterogeneity in populations, the importance of chromatin regulation other than gene-coding regions, and RNA modifications, which were not discussed in this paper, are also important future aspects. The possibility of achieving higher therapeutic efficacy is also discussed if epigenomic regulation can be understood and manipulated not only in tumor cells but also in the tumor microenvironment [42].

Epigenomic regulation is a complex process. However, hematology-oncology is leading the field of disease epigenetics, with new mechanisms being published and effective therapies developed one after another. In developing new therapeutic agents for hematopoietic tumors with underlying epigenomic abnormalities, it is important to ask what kind of epigenomic abnormalities the target disease display, what the causes of the epigenomic abnormalities are, and how we could reverse epigenomic abnormalities. These questions will pave the way of new treatments for intractable hematological diseases.
